# Critical shoulder angle (CSA): age and gender distribution in the general population

**DOI:** 10.1186/s10195-022-00627-w

**Published:** 2022-02-14

**Authors:** S. Gumina, G. Polizzotti, A. Spagnoli, S. Carbone, V. Candela

**Affiliations:** 1 Department of Anatomical, Histological, Forensic Medicine and Orthopaedics Sciences, Istituto Clinico Ortopedico Traumatologico (ICOT), Sapienza University of Rome, Latina, Italy; 2 Department of Public Health and Infectious Diseases, Sapienza University of Rome, Rome, Italy; 3 Orthopaedics and Traumatology Unit, San Feliciano Hospital, Rome, Italy; 4 Department of Anatomy, Histology, Legal Medicine and Orthopedics, University of Rome, Piazzale Aldo Moro 5, 00185 Rome, Italy

**Keywords:** Critical shoulder angle, Rotator cuff tear etiology, Rotator cuff tear risk factors, Extrinsic factors for rotator cuff tear, Scapular anatomy, Glenoid inclination

## Abstract

**Objective:**

Anatomical parameters and pathologies that can affect the critical shoulder angle (CSA) are subjects of discussion. To date, we do not know if the CSA value changes in the different decades of life in a population characterized by the same ethnicity, nor if there are differences related to gender or side. This study hypothesizes that age and gender may affect the CSA.

**Methods:**

Patients older than 15 years old affected by a shoulder trauma and who were discharged with a diagnosis of shoulder contusion were enrolled. A true AP view of the shoulder was obtained as well as data regarding age and gender of all participants. The CSA was measured by three authors, and interoperator reliability was assessed. Eight subcategories, according to decades of life, were considered. Finally, the studied population was divided into three subcategories according to CSA values (< 30°; 30–35°; ≥ 35°).

**Results:**

The initial sample comprised 3587 shoulder X-rays. The interobserver reproducibility was high, with an intraclass correlation coefficient of 0.865 (95% CI 0.793–0.915).

Two thousand eight hundred seventy-three radiograms were excluded. The studied group comprised 714 patients [431 females, 283 males; mean age (SD): 47.2 (20.9) years, range: 11–93 years]. The mean CSA was 33.6° (range: 24–50°; SD: 3.9°). The mean CSA values in females and males were 33.7°and 33.5°, respectively. The mean CSA values of the right and left shoulders were 33.3° and 33.9°, respectively (*p* > 0.05). Linear regression analysis showed a CSA increase by 0.04° every year. The mean CSA in subjects aged between 15 and 19 years was significantly lower than all the other groups, except for patients older than 80 years.

No significant differences were found between CSA subcategories, gender, or side.

**Conclusions:**

In the general population, the mean CSA value was 33.6°. No significant differences were found regarding the mean CSA value according to gender or side. A significant positive linear correlation between CSA and age was detected. In each decade of life, the CSA value, which is genetically determined, shows a large variability.

Level of evidence: IV.

## Introduction

The critical shoulder angle (CSA) corresponds to the angle obtained by the conjunction between two lines: the first line is drawn by joining the superior and inferior bony margins of the glenoid; the second line joins the inferior bony margin of the glenoid to the most lateral border of the acromion. Originally described by Moor et al. [1], the measure of the angle is carried out on radiograms in true AP view [1–7].

Anatomical parameters that can affect the angle width are the lateral acromial offset and the glenoid inclination. A more lateral acromial offset determines a more lateral deltoid origin and, biomechanically, results in more significant shear and lesser compressive vector of the deltoid across the glenohumeral joint [8]. A larger acromial index, which analyzes the lateral acromial offset, has been associated with rotator cuff disease [5, 7, 9–11]. Moreover, a high CSA has been indicated to be significantly related to the risk of cuff retear after repair [12–14]. A more upward-facing glenoid increases the risk for superior humeral translation, and may play a role in the development of rotator cuff degeneration and tear [3, 4, 15–19]. Furthermore, in 2013, Moor et al. [1] stated that patients with primary glenohumeral osteoarthritis have a significantly smaller CSA compared with controls and patients with rotator cuff tear. This hypothesis has been recently confirmed: CSA has been shown to be significantly different when patients with osteoarthritis are compared with subjects without degenerative changes of the glenohumeral joint [3, 6, 11]. Finally, isolated types II–IV superior labrum anterior to posterior lesions have been associated with a low CSA (< 30°) [10].

Although in three recent studies [20–22], the correlation of CSA with rotator cuff diseases has been strongly criticized, the popularity of CSA has increased over time. Nonetheless, many points on CSA remain to be elucidated [23]. In a retrospective analysis of longitudinally collected data, Chalmers et al. [24] stated the following: (1) most radiographs are of low quality for CSA measurement; (2) patients with cuff tear have higher CSA values than controls, but the difference is so small that it could be influenced by measurement error; (3) CSA is not correlated with cuff tear size; (4) CSA does not seem to change with time. Cabezas et al. [25], evaluating computed tomography (CT) reconstructions of 92 North American and 58 East Asian patients, observed that in the Asian group, the length of the acromion from the glenoid was statistically more significant compared with that registered in the Americans, by an average of 3.6 mm, which translated into a larger mean CSA.

To date, we do not know if the CSA changes according to gender, age, or side. To clarify these questions, we measured the CSA value on radiographs of a large number of subjects characterized by the same ethnicity, of different ages and gender. Our hypothesis is that age and gender may influence CSA.

## Materials and methods

The digitally available shoulder X-rays of Caucasian patients older than 15 years admitted to our emergency room for a reported shoulder trauma and discharged with a diagnosis of shoulder contusion (ICD-9: 92300) between January 2010 and December 2019, were obtained. Chart review was performed to confirm the history of trauma.

Only Caucasian patients were included in the analysis. Radiological inclusion criteria were as follows: type A glenoid rim and type 1 coracoid overlap according to Suter–Henninger principles [26, 27]. Radiological exclusion criteria were as follows: signs of humeral head or glenoid malunion, or anatomical defects (glenoid hypoplasia/agenesia, os acromialis).

Data regarding age and gender of all participants were obtained.

All radiographs were taken with a beam-to-film distance of 1.1 m at 70 kVp and 63 mAs. True AP views were obtained with the patient’s shoulder rotated posteriorly by approximately 35–45°, such that the plane of the scapula was parallel to the cassette. The beam was directed tangentially to the glenohumeral joint, and upper arm rotation was neutral.

The medical imaging program Osirix DICOM viewer (Pixmeo SARL, Geneve, Switzerland) was used to assess the CSA in the true AP view, as initially described [1].

The CSA was measured on radiograms of every subject by three different authors, to assess interoperator reliability. Previous studies showed that CSA can be measured reliably on plain radiographs [2, 6, 22, 28].

Eight subcategories, according to decades, were considered. Finally, the studied population was divided into three subcategories according to CSA values (< 30°; 30–35°; ≥ 35°).

All participants signed an informed consent form in accordance with the Declaration of Helsinki.

According to the law of our country, this study does not need any ethics committee approval.

## Statistical analysis

Patient characteristics were described using mean and standard deviation (SD) for continuous variables and percent for dichotomous variables. A plot of means and 95% confidence intervals was used to represent continuous variables. Comparisons between groups were performed by Chi-square test. One-way analysis of variance (ANOVA) followed by Bonferroni correction for multiple comparisons or Student *t*-test was used to compare CSA means between groups. Linear regression analysis was used to evaluate the relationship between CSA and age. All *p*-values were two sided, and a *p* < 0.05 was considered to be significant. All analyses were performed using software R version 3.6.1

## Results

**Fig. 1:**
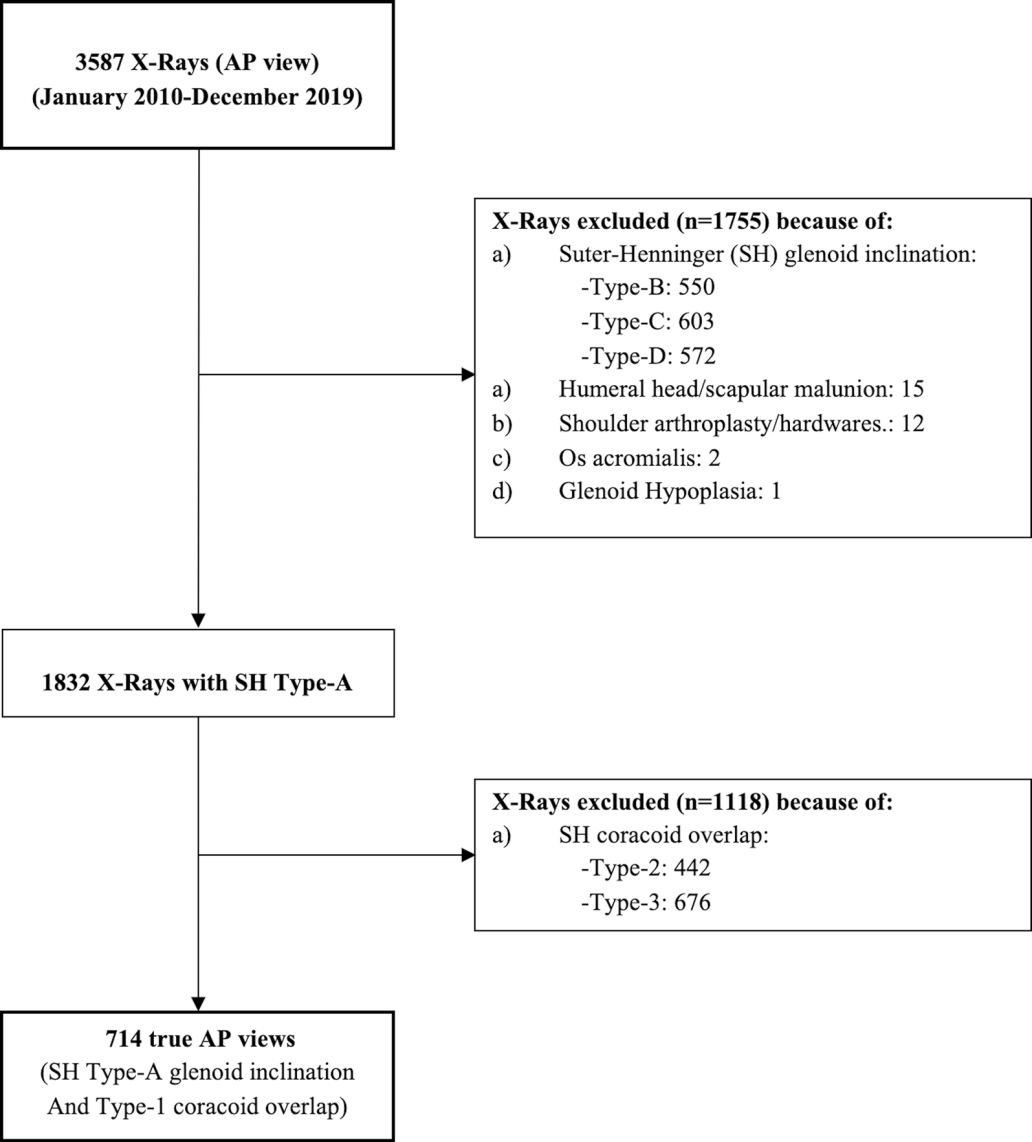
Flowchart of the study

The initial sample was composed of 3587 shoulder X-Rays. Two thousand eight hundred seventy-three radiograms were excluded (mean age: 48.7; SD 22.6; range: 16–83 years) from the analysis since one or more exclusion criteria were present (Fig. 1).

The studied group was finally composed of 714 [431 females, 283 males; mean age (SD): 47.2 (20.9) years].

X-rays of the right side were present in 423 cases (59.2%).

The interobserver reproducibility was high, with an intraclass correlation coefficient value of 0.865 (95% CI 0.793–0.915).

The mean CSA was 33.6° (SD: 3.9°; range: 24–50°).

The mean CSA values in females and males were 33.7° (SD: 3.9°) and 33.5° (SD: 3.9°), respectively. No significant differences were found.

The mean CSA values of the right and left shoulders were 33.3° (SD: 4.0°) and 33.9° (SD: 3.9°), respectively.

A significant relationship was found between CSA and age. In particular, CSA was found to increase by 0.04 every year (p < 0.0001).

**Fig. 2:**
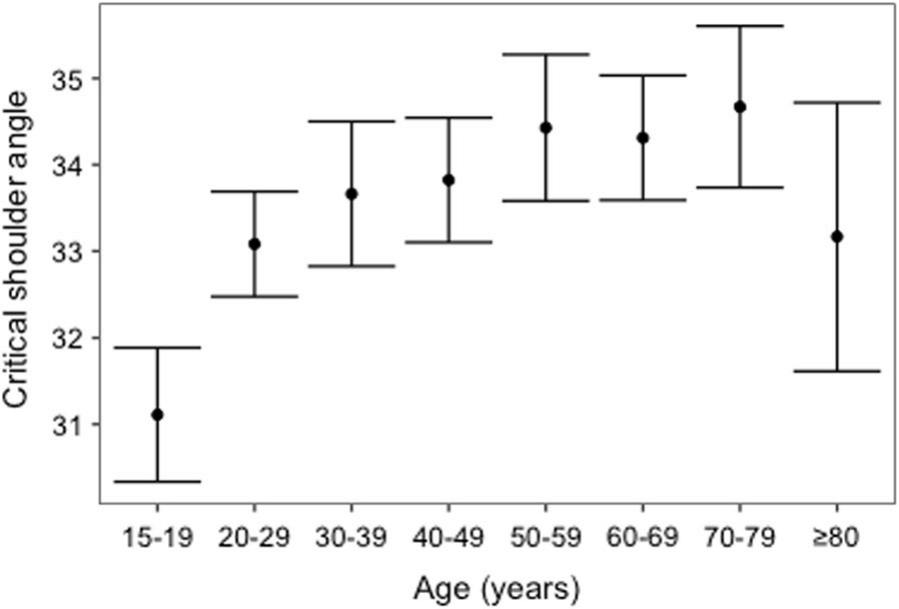
CSA values according to the different age groups. Means and 95% confidence intervals by age groups

According to the different age-groups, the mean CSA is shown in Fig. 2. Patients were equally distributed in each group. The mean CSA in the group of subjects aged between 15 and 19 years was significantly lower than all the other groups, except for patients older than 80 years (p < 0.001 for each comparison). No significant differences were found between the remaining age groups.

**Fig. 3:**
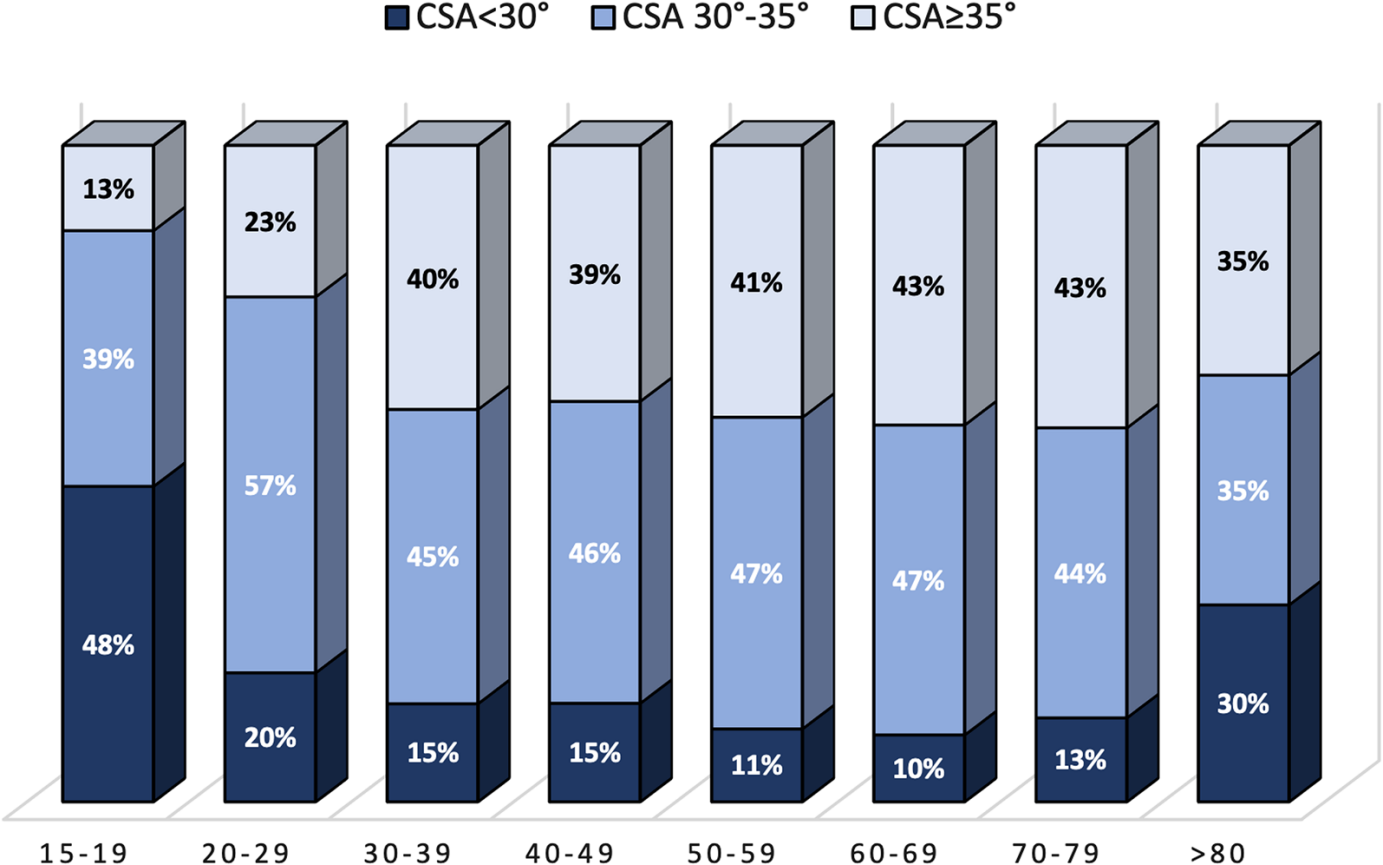
Distribution of CSA in the different age groups according to the three subcategories of CSA values (< 30°; 30–35°; ≥ 35°)

**Fig. 4:**
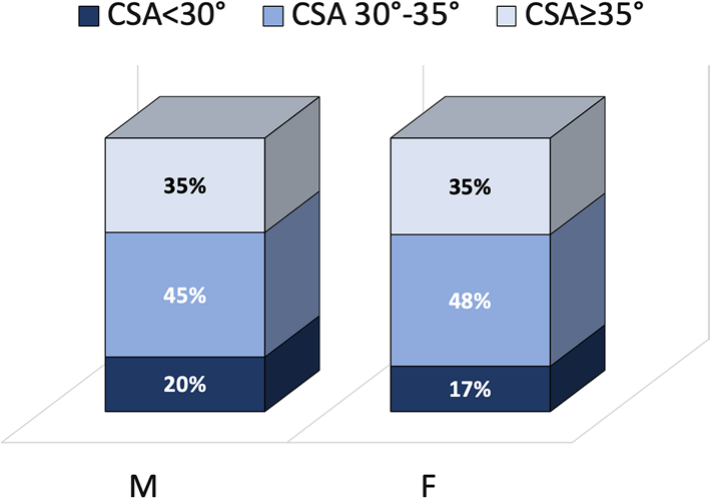
Distribution of CSA in males and females according to the three subcategories of CSA values (< 30°; 30–35°; ≥ 35°)

The distribution of CSA in the different age groups according to the three CSA subcategories (< 30°; 30–35°; ≥ 35°) is shown in Fig. 3; the distribution of CSA in females and males, according to the three subcategories is shown in Fig. 4. No significant differences were found between CSA subcategories, gender, and side.

## Discussion

The present study demonstrates the large variability of CSA among the general population. No significant differences were found regarding the mean CSA value by gender or side. However, a significant positive linear correlation between CSA and age was detected.

Recently, tendon degeneration (age-related or degeneration induced by genetics or medical conditions) seems to be the most credited theory for the onset of cuff tear [29–31]. The origin of rotator cuff tear is represented by an area of the tendon within few millimeters of its insertion characterized by relative hypovascularity [32–34]. Microvascularization may worsen due to hypertension [35], lung and other cardiovascular diseases [36], obesity [37], diabetes [23, 38], alcohol and smoking habits [39, 40].

The potential importance of the skeletal anatomy on the genesis of rotator cuff tear has been studied for decades, and remains a controversial subject [41–43]. The association between the acromion index with the development of cuff tears and glenohumeral osteoarthritis has been well analyzed [44]. In 2013, Moore et al. [1] developed a radiological parameter (CSA) that took into account the glenoid inclination and the acromion index, and hypothesized that this angle would correlate with the wear of cuff tendon and glenohumeral cartilage. Since then, a multitude of scientific papers focusing on CSA has enriched the scientific literature relating to the genesis of rotator cuff tear and glenohumeral arthritis. Although popularity and diffusion of CSA are increasing over time, at present we do not know if its value changes in a population characterized by the same ethnicity, nor if there are gender or side variations in the different decades of life. This is the first study to address these aspects.

Of the initial 3587 X-rays, only 19.9% of the radiographs met the Suter–Henninger criteria [27] for CSA measurement. The rate of available examinations is meager, even though our emergency room is exclusively dedicated to trauma and our specialized radiology technicians have been trained to perform the shoulder trauma series containing a true AP view. This is partly attributable to the scarce collaboration offered by the injured patient. The percentage is, in any case, similar to that reported by Chalmers et al. [24].

No significant differences were found regarding the mean CSA value per gender and side. This could mean that shoulder overuse induced by (a) a greater aptitude for practicing heavy work and sports (in men) or (b) side dominance, is not able to modify the acromion morphology or the glenoid inclination.

We analyzed the studied population according to different decades: a significant positive linear correlation between CSA and age was detected; however, the increase per year is about three-hundredths of a degree, showing that CSA is almost steady from the age of 30 years. This was confirmed by the fact that no significant differences in the mean CSA were found between the decades.

Moor et al. [1] stated that CSA value of healthy shoulders ranges from 30–35°: angles > 35° are associated with a high prevalence of rotator cuff tears; whereas shoulders with a CSA of < 30° are likely to be osteoarthritic. From a considerable number of scientific papers [1, 6, 24, 26, 27, 45–53], we identified that the mean CSA belonged to subjects with healthy shoulders (range from 30° to 37.4°) and with rotator cuff tear (range from 33.4° to 39.8°), respectively. The mean value of our 714 examined subjects was 33.6°. Our data is very reliable with regards to the general population because it was extracted from a large sample. However, it is still not comparable with data of the other series as our studied population could include patients with cuff tears and osteoarthritis.

In our series, the mean CSA value, registered in the different age groups, ranged between 31.1° and 34.7°. However, we observed that in each examined decade, there is a large variability (min: 24°; max: 50°).

It is unthinkable that, during the first decades of life, risk factors have occurred that are able to change the acromion offset or glenoid inclination. Therefore, it is plausible that CSA is genetically determined. This hypothesis is supported by the study of Gumina et al. [41] on elderly monozygotic and dizygotic twins, which observed that the anatomical features that influence the width of the subacromial space are mainly genetically determined.

The lowest mean values were observed in the population aged 15–20 years. It is plausible that an ossification center that is not perfectly fused, might affect the final CSA value.

This data allow us to come to two practical considerations: (1) whether in the third or fourth decade, a patient with a low CSA value will not have a CSA value predisposing to rotator cuff tear in the future and (2) in the third or fourth decade, patients could have a CSA value predisposing them to cuff tear; however, in this age group, the percentage of CSA > 35° is so high (23% in the 20s and 40% in the 30s) compared with the percentage of rotator cuff tears observed in their peers (2.5% in 20s and 6.7% in 30s), suggesting that this risk factor does not suddenly act. Longitudinal evaluation of these subjects will be decisive.

The CSA value is reliable, starting from the third decade. If the CSA value falls within the normal range (30–34°) in the third decade, it is plausible that it will remain in this range in the future.

It is possible to have a CSA value predisposing to cuff tear (> 35°) at the end of scapular skeletal maturity; however, as the percentage of subjects with CSA > 35° is high compared with the percentage of rotator cuff tears in their peers, it is conceivable that this risk factor does not suddenly act.

This study has some limitations: it is a retrospective review and useful information, such as the patients’ dominant side were not obtained. Furthermore, no longitudinal evaluation was performed.

## Conclusions

In the general population, the mean CSA value is 33.6°. No significant differences were found regarding the mean CSA value distinguished by gender or side. A significant positive linear correlation between CSA and age was detected. In each decade of life, the CSA value shows a large variability.
